# Constructing the public in public perceptions research: A case study of forest genomics

**DOI:** 10.1177/09636625231210453

**Published:** 2023-12-14

**Authors:** Valerie Berseth, Jennifer Taylor, Jenna Hutchen, Vivian Nguyen, Stephan Schott, Nicole Klenk

**Affiliations:** Carleton University, Canada; University of Toronto, Canada; Carleton University, Canada; University of Toronto Scarborough, Canada

**Keywords:** attitudes on genetics, co-production of knowledge, deficit model, imagined publics, risk, science attitudes and perceptions, scientific controversies, technology assessment

## Abstract

Contemporary scientific and technological endeavours face public and political pressure to adopt open, transparent and democratically accountable practices of public engagement. Prior research has identified different ways that experts ‘imagine publics’ – as uninformed, as disengaged, as a risk to science, and as co-producers of knowledge – but there has yet to be a systematic exploration of how these views emerge, interact and evolve. This article introduces a typology of imagined publics to analyse how publics are constructed in the field of forest genomics. We find that deficit views of publics have not been replaced by co-production. Instead, deficit and co-productive approaches to publics co-exist and overlap, informing both how publics are characterized and how public perceptions are studied. We outline an agenda for deepening and expanding research on public perceptions of novel technologies. Specifically, we call for more diverse and complex methodological approaches that account for relational dynamics over time.

## 1. Introduction

Science and technology studies (STS) articulate the importance of understanding how scientists view publics (Besley and Nisbet, 2013; Davies, 2008; Heidenreich, 2015). Extensive critique has been levied against the idea that publics lack sufficient understanding of science and technology and that education is needed to remedy this perceived knowledge deficit (McNeil, 2013; Simis et al., 2016; Sturgis and Allum, 2004). Constructivist or co-productive approaches to public understanding of science take a contrasting view, arguing that publics have legitimate knowledge, concerns, and values and should be meaningfully included in decision-making about science and technology (Wehrens, 2014; Wyborn, 2015a). Publics may also be viewed as disengaged or disinterested in science (Burns and Medvecky, 2018), or as a risk that must be carefully managed (Welsh and Wynne, 2013). Despite sustained interest in these different constructions of publics, there have been few attempts to trace the frequency and impact of expert views of publics in published research.

Informed by the concept of ‘imagined publics’, this article examines how publics are defined and surveyed in research on public perceptions of new technologies. Using forest genomics as a case study, we ask: how are publics imagined and studied in forest genomics research, and what are the implications of different imaginings and methods of engagement with publics over new technologies? Genomic technologies for screening and retrieving genome-wide information are diverse, including DNA/RNA sequencing, transcriptomics, proteomics, metabolomics, phylogenomics, population and landscape genomics. These approaches have far-reaching and uncertain implications for forest management and, in some cases, test the boundaries of acceptable human intervention in managing natural processes.

We conducted a qualitative systematic literature review (Paré et al., 2015) to examine how research on genomics for forest management views or imagines ‘the public.’ STS studies are focused on how technology controversies are constructed (Chilvers et al., 2018), and whose evidence, arguments, and framings have authority in a contested domain (Jasanoff, 2004). Such controversies are constituted by diverse knowledges and publics, which may or may not share common assumptions or common consensus. Genomics is fertile terrain to scrutinize ‘expert’ knowledge claims and the uncertain, partial and contingent conditions under which they become accepted as facts (Whatmore and Landström, 2011). Gene technologies have been the subject of high-profile public controversies related to their perceived risks, uncertainties, and their potential to transgress natural boundaries (Aubin et al., 2011; Gottweis, 2005; Wynne, 2001). Past controversies, such as genetic modification, have contributed to public expectations that debates about emerging technologies will be open, transparent, and democratically accountable (Davies et al., 2022). However, increasing the amount of public engagement does not necessarily imply a transformation in the power relations that shape the production and governance of science and technology. Understanding expert claims about publics is therefore critical to informing debates about gene technologies and genomic science more broadly.

Our approach draws attention to peer-reviewed literature as an underappreciated site where expert accounts of publics are constructed. Published articles provide a record of how experts define their research and their field in relation to outside audiences. They also offer an opportunity to investigate how different views of publics affect how public perceptions are studied and represented. We argue that constructions of the public are not only meaningful for shaping engagement between science and society; they also shape the production of knowledge about public perceptions. Decisions about whose views to survey, which methods to use, which variables to analyse, and for what purpose, are predicated on normative assumptions about publics and their relation to science and technology.

Below, we outline the methodology and conceptual framework of imagined publics that guided this study. Our conceptual framework brings together different imagined publics referenced in STS literature in a typology of imagined publics, including views of the public as uninformed, as disengaged, as a risk and as co-producers of knowledge. We then present the results of the literature review, using this typology to analyse trends in methodological approaches, research streams and debates that have shaped the field of forest genomics. The discussion takes stock of these findings and advances an agenda for expanding and deepening research on public views of new technologies. We conclude by reflecting on the implications of imagining and studying publics for the science-society relationship, both within and beyond the field of forest genomics.

## 2. Imagined publics typology

The idea of an ‘imagined public’ refers to experts’ tendency to imagine the public as a collective, or multiple collectives, of individuals on the opposing side of an epistemic divide between science and society, and to ascribe certain ideas about their competencies (or lack thereof) (Maranta et al., 2003). Imagining publics is not only a cognitive exercise; it provides models that experts and decision-makers can use to structure when and how they interact with publics (Walker et al., 2010). While these constructions may bear little resemblance to individuals that exist in the world (Ellis et al., 2010), they inform expert practices, discourses and expectations.

**Figure 1.**
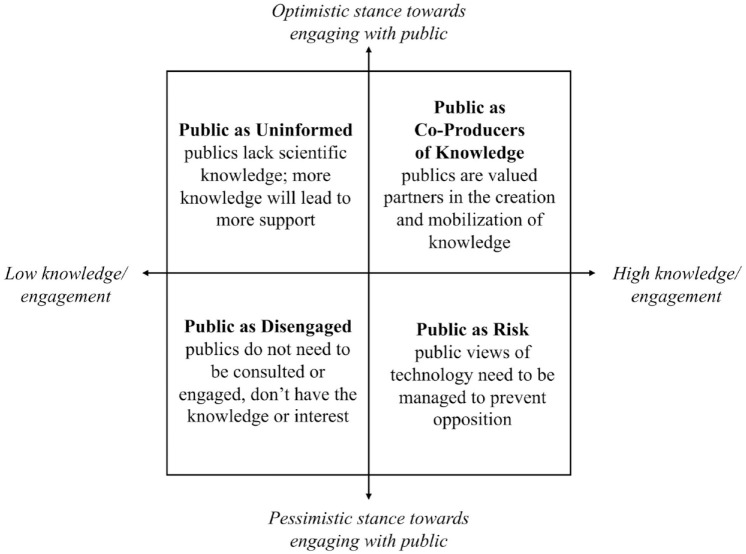
Typology of imagined publics.

Empirical research has found support for different ways that experts imagine publics – as passive recipients of scientific information, as instigators of opposition to novel technologies, as uninterested in science or as knowledge holders in their own right. These perspectives – further expanded below – were chosen because they differ in their orientation towards public knowledge and the value (or risks) in engaging with the public on matters of science and technology. We bring these together in a typology of imagined publics (Figure 1), which enables us to trace how different understandings of publics have emerged and evolved over time in the field of forest genomics.^1^ We summarize these in Figure 1, oriented along two axes. The horizontal axis represents the level of perceived knowledge and/or engagement with science that publics have. The vertical axis represents the polarity of views towards engaging with the public, from optimistic to pessimistic. While they are represented as distinct, these imaginaries may intersect. For example, publics may be viewed as risky because they are believed to be uninformed.

### Public as disengaged

Burns and Medvecky (2018) define the ‘disengaged’ public as lacking interest in science. The idea of a disengaged public arose from early science communication literature and was initially characterized by a normative assumption that it is desirable for the public to be informed about science, and a concern for how to effectively address the public’s lack of trust and interest in science (Burns and Medvecky, 2018). Some more recent literature problematizes this, arguing that it is in part experts’ misunderstandings and alienations of marginalized groups that contributes to divisions between science and the public (e.g. Dawson, 2018). The implicit negative assumptions about publics’ interest in science, coupled with the view that the disengaged are lacking knowledge, places it in the bottom left quadrant of Figure 1.

### Public as uninformed (deficit model)

The ‘public as uninformed’ view focuses on the amount the public knows (or does not know) about science and technology. From this perspective, the primary barrier to public support for science is lack of knowledge, and thus education is the solution. This view underlies the deficit model of the public, whereas Sturgis and Allum (2004) write, ‘. . .it is the public that are assumed to be “deficient”, while science is “sufficient”’ (p. 57). In contrast to the ‘public as disengaged’ imaginary, the deficit view is more likely to take a neutral or optimistic attitude, encouraging greater knowledge translation and one-way communication to educate the public and fill the deficit. For this reason, it is located in the top half of Figure 1.

### Public as risk

Although an informed public has typically been characterized as the desired outcome of science communication, some studies have found concern about engaged and informed (or, *mis*- or *under-*informed) publics. This view is rooted in an anxiety about public anti-science sentiment, and the idea that publics may pose threats to scientific progress (Hess, 2015; Lidskog, 2016; Welsh and Wynne, 2013). A central element of the ‘public as risk’ view is the management of public opinion through strategies such as withholding information or avoiding discussing controversial topics with the public. This perspective occupies the bottom right quadrant of Figure 1 because of its negative view towards an informed public.

### Public as co-producers of knowledge

Jasanoff (2004) describes co-production as the way that scientific knowledge ‘embeds and is embedded in social practices, identities, norms, conventions, discourses, instruments and institutions’ (p. 2). Foundational to co-productive imaginings of publics is a recognition that all knowledge claims are underpinned by tacit commitments to particular forms of social order, identities and relations (Wyborn, 2015b). Participatory and collaborative frameworks have been developed and implemented to foster co-production among users (of knowledge, technology, innovation) including transdisciplinary frameworks, and living laboratories, among others (Culwick et al., 2019; Polk, 2015). Because this co-production model understands publics as having valuable knowledge and encourages a high degree of engagement, it is located in the top right corner of Figure 1.

## 3. Methodology

**Figure 2.**
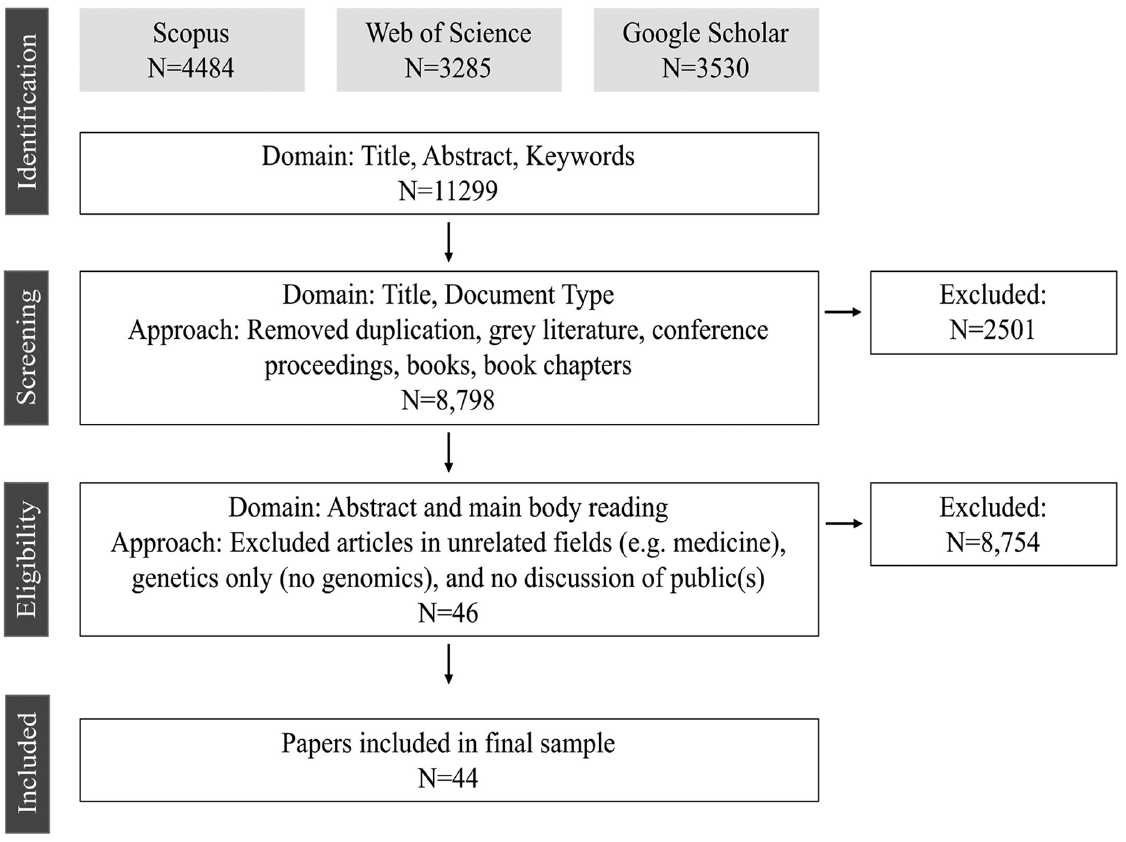
Literature search flow diagram.

We conducted a qualitative systematic literature search (Paré et al., 2015), which involves a comprehensive search of literature to explore narrowly defined research questions, uses qualitative and/or quantitative data analysis, and synthesizes findings through narrative summary and commentary or interpretation of the results. Two academic databases were used: Scopus and Web of Science (Figure 2). Search terms in **Scopus** were: TITLE-ABS-KEY((forest* OR ‘forest management’ OR ‘forest pests’) AND ((genomic* OR genetic* OR ‘genetic engineering’) AND (perception* OR ‘public perception*’ OR attitude* OR trust OR social OR cultural OR acceptance OR risk OR barrier*))); and **Web of Science** were: TS = ((forest* OR ‘forest management’ OR ‘forest pests’) AND ((genomic* OR genetic* OR ‘genetic engineering’) AND (perception* OR ‘public perception*’ OR attitude* OR trust OR social OR cultural OR acceptance OR risk OR barrier*))). We supplemented these with an additional search on Google Scholar. Because Google Scholar limits search string complexity, searches using the terms genomic* or genetic* were too broad to return relevant results. We narrowed the Google Scholar search to assisted migration,^2^ which had emerged as the most frequently cited application of genomics in forest management based on our initial screening of the Web of Science and Scopus results. The search string used in **Google Scholar** was: (‘forestry’ or ‘tree’) and ‘assisted migration’.

Articles were included if they were: (1) peer-reviewed (including articles, reviews, editorials, and essays/commentaries); (2) focused on genomics in forest management; (3) discussed some aspect of publics including acceptance, opposition or engagement, risk perception, public knowledge and public debate. The search resulted in 44 articles about genomics and public perceptions (Table 1). Further details on the sampled studies can be found in the Supplemental Material.

**Table 1.** Sampled studies.

Number	Citation
1	Aubin I, Garbe CM, Colombo S, et al. (2011) Why we disagree about assisted migration: Ethical implications of a key debate regarding the future of Canada’s forests. *The Forestry Chronicle* 87(06): 755–765.
2	Aucott M and Parker RA (2021) Medical biotechnology as a paradigm for forest restoration and introduction of the transgenic American chestnut. *Conservation Biology* 35(1): 190–196.
3	Bilodeau P, Roe AD, Bilodeau G, et al. (2019) Biosurveillance of forest insects: Part II – adoption of genomic tools by end user communities and barriers to integration. *Journal of Pest Science* 92(1): 71–82.
4	Blue G and Davidson D (2021) Co-producing uncertainty in public science: The case of genomic selection in forestry. *Public Understanding of Science* 30(4): 455–469.
5	Ćalić I, Bussotti F, Martínez-García PJ, et al. (2016) Recent landscape genomics studies in forest trees – What can we believe? *Tree Genetics & Genomes* 12(1): 3.
6	Chaves SF da S, Alves RM and Dias A dos S (2021) Contribution of breeding to agriculture in the Brazilian Amazon. I. Açaí palm and oil palm. 21(S): e386221 S8.
7	Crann SE, Fairley C, Badulescu D, et al. (2015) Soils, microbes, and forest health: A qualitative analysis of social and institutional factors affecting genomic technology adoption. *Technology in Society* 43: 1–9.
8	Cullingham CI, Janes JK, Hamelin RC, et al. (2019) The contribution of genetics and genomics to understanding the ecology of the mountain pine beetle system. *Canadian Journal of Forest Research* 49(7): 721–730.
9	Findlater K, Hagerman S, Kozak R, et al. (2022) Redefining climate change maladaptation using a values-based approach in forests. *People and Nature* 4(1): 231–242.
10	Findlater KM, St-Laurent GP, Hagerman S, et al. (2020) Surprisingly malleable public preferences for climate adaptation in forests. *Environmental Research Letters* 15(3): 034045.
11	Hagerman S, Satterfield T, Nawaz S, et al. (2021) Social comfort zones for transformative conservation decisions in a changing climate. *Conservation Biology* 35(6): 1932–1943.
12	Hajjar R, McGuigan E, Moshofsky M, et al. (2014) Opinions on strategies for forest adaptation to future climate conditions in western Canada: Surveys of the general public and leaders of forest-dependent communities. *Canadian Journal of Forest Research* 44(12): 1525–1533.
13	Hamelin RC (2012) Contributions of genomics to forest pathology. *Canadian Journal of Plant Pathology* 34(1): 20–28.
14	Hamelin RC and Roe AD (2020) Genomic biosurveillance of forest invasive alien enemies: A story written in code. *Evolutionary Applications* 13(1): 95–115.
15	Han Q, Keeffe G, Caplat P, et al. (2021) Cities as hot stepping stones for tree migration. *npj Urban Sustainability* 1(1): 12.
16	Harfouche A, Meilan R and Altman A (2011) Tree genetic engineering and applications to sustainable forestry and biomass production. *Trends in Biotechnology* 29(1): 9–17.
17	Hazarika R, Bolte A, Bednarova D, et al. (2021) Multi-actor perspectives on afforestation and reforestation strategies in Central Europe under climate change. *Annals of Forest Science* 78(3): 60.
18	Hewitt N, Klenk N, Smith AL, et al. (2011) Taking stock of the assisted migration debate. *Biological Conservation* 144(11): 2560–2572.
19	Homyack J, Sucre E, Magalska L, et al. (2022) Research and Innovation in the Private Forestry Sector: Past Successes and Future Opportunities. *Journal of Forestry* 120(1): 106–120.
20	Hope ES, Barsi DC and McKenney DW (2017) Assessing the adoption and impact of genomics research at the Canadian Forest Service. *The Forestry Chronicle* 93(02): 118–121.
21	Huang Y, Zhen Z, Cui Z, et al. (2021) Growth and arthropod community characteristics of transgenic poplar 741 in an experimental forest. *Industrial Crops and Products* 162: 113284.
22	Isabel N, Holliday JA and Aitken SN (2020) Forest genomics: Advancing climate adaptation, forest health, productivity, and conservation. *Evolutionary Applications* 13(1): 3–10.
23	Jepson P and Arakelyan I (2017) Exploring public perceptions of solutions to tree diseases in the UK: Implications for policy-makers. *Environmental Science & Policy* 76: 70–77.
24	Keriö S, Daniels HA, Gómez-Gallego M, et al. (2020) From genomes to forest management – tackling invasive *Phytophthora* species in the era of genomics. *Canadian Journal of Plant Pathology* 42(1): 1–29.
25	Lavrik M (2021) Constructing regulation on assisted migration: Findings from science and ethics. *SN Social Sciences* 1(9): 242.
26	Maruta AA, Boxall P and Mohapatra S (2018) Heterogeneity in attitudes underlying preferences for genomic technology producing hybrid poplars on public land. *Canadian Journal of Forest Research* 48(8): 869–880.
27	Moshofsky M, Gilani HR and Kozak RA (2019) Adapting forest ecosystems to climate change by identifying the range of acceptable human interventions in western Canada. *Canadian Journal of Forest Research* 49(5): 553–564.
28	Neale DB (2007) Genomics to tree breeding and forest health. *Current Opinion in Genetics & Development* 17(6): 539–544.
29	Neale DB and Kremer A (2011) Forest tree genomics: Growing resources and applications. *Nature Reviews Genetics* 12(2): 111–122.
30	Neff MW and Larson BMH (2014) Scientists, managers, and assisted colonization: Four contrasting perspectives entangle science and policy. *Biological Conservation* 172: 1–7.
31	Nilausen C, Gélinas N and Bull G (2014) Proposed research on social perception of marker-assisted selection and its role in the forests of British Columbia. *The Forestry Chronicle* 90(05): 666–669.
32	Nilausen C, Gélinas N and Bull G (2016) Perceived Acceptability of Implementing Marker-Assisted Selection in the Forests of British Columbia. *Forests* 7(12): 286.
33	Pelai R, Hagerman SM and Kozak R (2020) Biotechnologies in agriculture and forestry: Governance insights from a comparative systematic review of barriers and recommendations. *Forest Policy and Economics* 117: 102191.
34	Pelai R, Hagerman SM and Kozak R (2021a) Seeds of change? Seed transfer governance in British Columbia: Insights from history. *Canadian Journal of Forest Research* 51(2): 326–338.
35	Pelai R, Hagerman SM and Kozak R (2021b) Whose expertise counts? Assisted migration and the politics of knowledge in British Columbia’s public forests. *Land Use Policy* 103: 105296.
36	Peterson St-Laurent G, Hagerman S and Kozak R (2018) What risks matter? Public views about assisted migration and other climate-adaptive reforestation strategies. *Climatic Change* 151(3–4): 573–587.
37	Peterson St-Laurent G, Hagerman S, Findlater KM, et al. (2019) Public trust and knowledge in the context of emerging climate-adaptive forestry policies. *Journal of Environmental Management* 242: 474–486.
38	Peterson St-Laurent G, Kozak R and Hagerman S (2021a) Cross-jurisdictional insights from forest practitioners on novel climate-adaptive options for Canada’s forests. *Regional Environmental Change* 21(1): 4.
39	Peterson St-Laurent G, Oakes LE, Cross M, et al. (2021b) R–R–T (resistance–resilience–transformation) typology reveals differential conservation approaches across ecosystems and time. *Communications Biology* 4(1): 39.
40	Plomion C, Bastien C, Bogeat-Triboulot M-B, et al. (2016) Forest tree genomics: 10 achievements from the past 10 years and future prospects. *Annals of Forest Science* 73(1): 77–103.
41	Porth I, Bull G, Ahmed S, et al. (2015) Forest genomics research and development in Canada: Priorities for developing an economic framework. *The Forestry Chronicle* 91(01): 60–70.
42	Sork VL, Aitken SN, Dyer RJ, et al. (2013) Putting the landscape into the genomics of trees: Approaches for understanding local adaptation and population responses to changing climate. *Tree Genetics & Genomes* 9(4): 901–911.
43	Thiffault N, Raymond P, Lussier J-M, et al. (2021) Adaptive Silviculture for Climate Change: From Concepts to Reality Report on a symposium held at Carrefour Forêts 2019. *The Forestry Chronicle* 97(01): 13–27.
44	Touchette L, Beaudoin J-M, Isabel N, et al. (2021) How to put forest and conservation genomics into motion for and with Indigenous communities? *The Forestry Chronicle* 97(3): 300–314.

We employed a directed content analysis (Hsieh and Shannon, 2005), starting with four initial coding categories identified from literature on the science-society relationship and scientists’ understandings of the public: *public as uninformed (deficit model), public as disengaged, public as risk, and public as co-producer.* While these themes are commonly referenced in literature on science and society, they are not an exhaustive list of ways to conceptualize publics, so the data were re-read to identify any constructions of publics that were not captured in the typology.

Coding proceeded in three stages. First, JT coded article meta-data (year, title, journal, article type, research objectives, authorship), content (research objectives, publics surveyed, methods, findings), and framing of genomics (approaches discussed, arguments supporting, arguments against genomics, barriers to genomics uptake). Second, VB coded any reference to publics according to the four coding categories described above. Any passages that did not fall into one of the four categories was assigned a new code. Authors VB, JT and NK met regularly to review coding and resolved differences through negotiation. Third, to better understand how publics are characterized in this field, VB re-read the coded passages and generated subcodes from emergent themes. These subcodes were then organized into a thematic network (Attride-Stirling, 2001) including the goals of engaging with publics, barriers or problems to public engagement, proposed solutions, and responsibility for public engagement (Table 2).

**Table 2.** Frequencies of coded themes in forest genomics literature, N(%), calculated as percentage of articles within each type of imagined public (uninformed, disengaged, risk, co-producer).

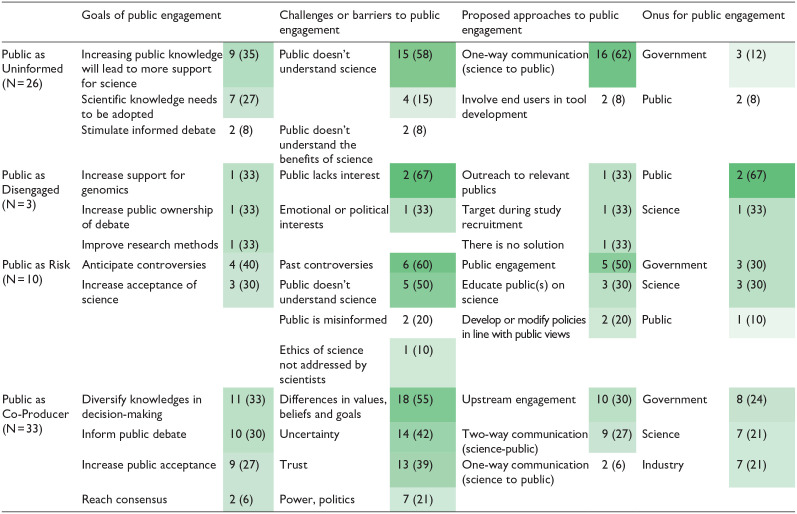

Cells are shaded to demonstrate frequencies, with darker shades corresponding to higher percentage of articles in which a theme appeared.

## 4. Results

### Major trends in forest genomics

**Figure 3.**
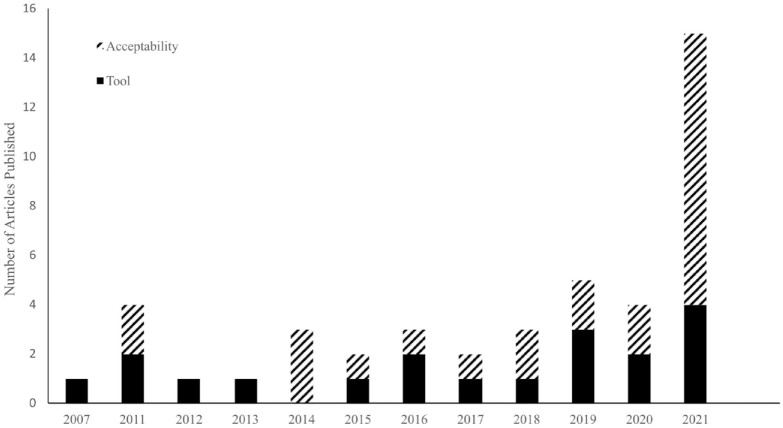
Sampled articles published by year and by research stream.

Forest genomics research that includes consideration of public perceptions has grown steadily since 2007, with a substantial increase in 2021 (Figure 3). This research has followed two trajectories, each of which is driven by different goals that inform their respective orientations towards publics. The first stream (N = 19) comprised articles that *promoted genomic tools*. These studies described the contribution of genomics to forest studies and highlighted advancements as well as future prospects (e.g. Studies 13, 29, 40 in Table 1). There was little discussion of who would benefit from these tools. Where publics were mentioned, end users and stakeholders in forest management (e.g. tree breeders, forest managers) were the primary focus. These studies also aimed to identify facilitators and barriers to genomics uptake, citing public lack of genomic knowledge, relevance, efficiency, and cost as the primary obstacles (e.g. Studies 3, 6, 7, 14, 43 in Table 1), in addition to technical obstacles (e.g. Studies 8, 13 in Table 1).

The second stream (N = 25) focused on the *acceptability of genomics*. This interest in acceptability led studies in this stream to directly engage with and survey public perceptions of novel genomic technologies. Publics surveyed ranged widely, from the general public to forest managers, environmentalists, forestry industry professionals, Indigenous peoples and forest communities. Studies typically sought to determine factors affecting the acceptability of genomic applications, such as demographics (e.g. Study 12 in Table 1), and comparative studies across different regions (e.g. Studies 11, 38 in Table 1). This category also included studies of perceived uncertainties (e.g. Study 4 in Table 1), genomics debates (e.g. Studies 18, 30 in Table 1), and the malleability of public preferences (e.g. Studies 10, 12 in Table 1).

### Imagined publics in genomics research

We found frequent representation of *public as uninformed* (N = 26) and *public as co-producer* (N = 33) in the literature surveyed. There were almost no references to the *public as disengaged* (N = 3), but nearly one-quarter of the articles contained references to *public as risk* (N = 10). These categories are not mutually exclusive, and some articles referenced more than one view of publics. Table 2 displays the results from thematic coding of articles in each category.

#### Public as uninformed

Articles that referred to the public as uninformed (N = 26) generally shared the view that the public has a limited understanding of science, and that lack of knowledge is (or could be) a barrier to the uptake of genomics in forest management. This argument overlapped with another assertion: that publics are not aware of, or do not understand, the benefits of genomic science. Few articles expounded on the theoretical basis for the link between knowledge and acceptability, although two (Studies 3, 7 in Table 1) referenced the Diffusion of Innovations Theory, which posits that innovations need to be compatible with existing knowledge and easy to understand.

Yet, despite how frequently it was mentioned, evidence for a correlation between knowledge and acceptability was inconclusive. In several articles, respondents self-reported a lack of familiarity with genomics and its applications (Studies 7, 17, 32 in Table 1). Maruta et al. (2018) found a statistically significant difference between the cluster of ‘Knowledgeable’ respondents and other clusters in support of adopting novel technologies. Findlater et al. (2020) also found that people with more knowledge of forestry reported a slight increase in support for assisted migration outside of natural ranges when given new information. Conversely, Peterson St-Laurent et al. (2018, 2019) did not find a significant relationship between knowledge and support for genomic policies and interventions. Nilausen et al. (2016) found differences in support for marker-assisted selection^3^ across three groups (industry, government, and environmental organizations) but did not report a significant relationship between acceptance and education or genomics knowledge.

The most frequently cited goal of public engagement in this category was improving the likelihood of adoption through one-way translation of complex genomic knowledge into lay terms. For example, multiple studies promoted the creation of educational packages or materials (Studies 3, 31, 32, 44 in Table 1) or user-friendly tools (Studies 14, 28 in Table 1). However, studies rarely discussed who bears the responsibility for this knowledge transfer, with some references to government and publics (Studies 7, 26 in Table 1) or high-level depictions of a linear path from research to implementation (Studies 16, 20 in Table 1).

#### Public as disengaged (N = 3)

Only three articles described the public as disengaged. Neff and Larson (2014) and Peterson St-Laurent et al. (2018) point to lack of interest among publics in science on assisted colonization and climate change adaptation, respectively. Blue and Davidson (2021) found an assumption among some forest genomics experts that disinterested publics are unwilling to engage with science because of emotional (e.g. fear of technology) or financial (e.g. donations to ENGOs) interests in opposing technological development. Overall, the sample size was too small to identify meaningful patterns in the data.

#### Public as risk (N = 10)

The depiction of *public as risk* was associated with concerns about potential social controversies over genomic technologies. Nearly two-thirds of articles in this category specified past controversies over genetically modified organisms (GMOs) (Studies 23, 26, 33, 37 in Table 1) and more contentious forms of assisted migration (Study 27 in Table 1) as potential barriers to uptake of genomics in forest management. There was concern that genomic applications could be mistaken for GMOs and experience the same lack of public trust shown towards genetics and forest management more broadly (Studies 33, 37 in Table 1).

These articles aimed to anticipate and mitigate potential controversies surrounding novel genomic technologies and to increase support for adoption. The foremost recommendation was conducting public outreach before implementation, while a minority promoted improving the image of genomics through public debate (Study 2 in Table 1), marketing (Study 26 in Table 1), and seeking positive media coverage (Study 27 in Table 1). However, Pelai et al. (2020) cautioned that upstream efforts to simply educate publics are unlikely to succeed, and that tokenistic responses to public opposition neglect fundamental concerns and values that underly these controversies. Responsibility for managing public opposition was attributed to government and scientists in approximately half of the articles in this group.

#### Public as co-producer (N = 33)

The most prevalent depiction of publics was *public as co-producer.* However, this is a relatively recent trend, with two-thirds of articles in this category published after 2018 and driven by a rise in articles in the ‘acceptability’ stream. Articles varied widely in terms of the depth and nature of their engagement with ideas typically associated with a co-production approach. Most briefly discussed values, uncertainty, trust and power dynamics. A minority (N = 5) took a stronger co-production approach, critiquing the dominant normative order that privileges scientific knowledge or discussing co-production by name (Studies 4, 25, 34, 35, 44 in Table 1).

Contending with multiple values, beliefs, and worldviews was the most commonly cited challenge to meaningful public engagement in this category, and articles generally supported the idea that values influence views of forest management and the perceived acceptability of genomic tools (e.g. Studies 9, 10, 11, 25 in Table 1). Some tested this effect directly. For example, Moshofsky et al. (2019) found that individualist worldviews were associated with higher preferences for more controversial, high-risk scenarios than egalitarian or hierarchist views. Other studies (e.g. Studies 9, 10, 11 in Table 1) found that despite the importance of values, preferences are malleable and subject to change.

Co-production articles featured the most wide-ranging objectives, divided roughly evenly between diversifying the knowledges used in decision-making, supporting public debate and seeking public acceptance for genomic technologies. Proposed solutions were split between 30% promoting upstream engagement and 27% supporting two-way communication between scientists and publics. Responsibility was likewise split among government, science and industry.

#### Emergent categories: Public as consumer, public as owner

Two additional imagined publics emerged through coding. The first, *public as consumer* (N = 5) characterized the audience for forest genomics applications as stakeholders or end-users that would support adoption if genomics provided economic gains or if technologies were cost-effective. A second imaginary specific to Canadian studies of *public as owner* (N = 4) described forests in Canada as ‘publicly owned’ (Study 41 in Table 1) and governments as forest ‘custodians’ (Study 38 in Table 1).

### Methodologies informing public perception research

We found differences between studies of public perception based on their approaches to publics described above. These differences related to the methods used, the rationale for studying public perceptions, and the way the studies treated scientific and non-scientific knowledge. Because the objectives of the ‘tools’ stream were to promote genomic tools and increase uptake, these articles largely did not survey publics directly and comprised the majority of articles coded as ‘uninformed’, but not ‘co-producer’. One exception is Nilausen et al. (2014) who proposed studying acceptability by providing participants with ‘a brief educational package’ on genomic selection before interviews (p. 668).

Of the 19 articles coded as both ‘uninformed’ and ‘co-producer’, 10 surveyed publics directly. The most frequent method for studying perceptions was quantitative surveys (N = 9), with two studies employing a mixed-methods approach that combined surveys with interviews (Study 32 in Table 1) or focus groups (Study 23 in Table). Only one study employed qualitative interviews as the sole methodology (Study 7 in Table 1). Across these articles, there was moderate to strong normative support for the acceptance of genomic science and technologies. For example, Jepson and Arakelyan (2017) sought to ‘provide science and policy with an “upstream steer” . . . to increase the acceptability of policy’ related to genomic-informed tree breeding (p. 71). Content-specific knowledge about forest ecology, management, and genomics was treated as an independent variable affecting acceptability in the majority of articles (N = 8), either asking respondents to self-report knowledge (Study 17 in Table 1) or directly testing and fact-checking respondent knowledge (Studies 10, 26, 32, 36, 37 in Table 1).

Finally, articles that were coded as ‘co-producer’ but not ‘uninformed’ took a more qualitative and open-ended approach to studying public perceptions (N = 7). Methods included interviews (Studies 4, 34, 35 in Table 1), focus groups (Study 9 in Table 1), Q-surveys, and online surveys (Studies 11, 36 in Table 1). Instead of assessing public knowledge as an independent variable, these studies examined a wide range of other factors that affect acceptability, including values and trust. Articles in this group also surveyed experts in genomics and forestry to understand how expertise informs decision-making about policy, risk and uncertainty (Studies 4, 34, 35 in Table 1). There was a normative aim to seek input from knowledge holders outside Western science, with little to no stated support in the articles for facilitating technology adoption. For example, Hagerman et al. (2021) investigated how values inform public perceptions to support ‘scholars and policy makers . . . to engage robustly in conversations about novel and transformative interventions’ (p. 1934). Neff and Larson (2014) stated that ‘. . .the debate over assisted colonization has largely been framed by academic conservation scientists, so the views of other stakeholders remain underrepresented’ (p. 2).

## 5. Discussion

There has been a steep increase in the number of studies examining public perceptions of forest genomics and it may be reasonable to suggest that there may be a ‘co-production turn’ emerging in genomics research. By analyzing forest genomics literature through a novel typology of imagined publics, we have shown that fundamental differences in orientations to genomics – as tools to be implemented or as objects of public scrutiny and potential (in)acceptability – are associated with different conceptualizations of publics and the science-society relationship. In articles that explicitly seek to promote genomic tools, minimal engagement with publics and the presence of deficit views are unsurprising. However, a closer examination of how publics are characterized in articles seeking to explain the acceptability of genomic technologies reveals a more complex picture. First, ideas not commonly associated with co-production, such as support for one-directional science communication, were frequently present in articles that employ concepts and terminology from co-production. This suggests that notions of ‘publics as lacking knowledge’ and ‘science as educator’ are still present in this field and that different ways of imagining publics can overlap. For example, research promoting upstream engagement may argue for involving publics earlier in decision-making, but still depict a linear process from laboratory to society (Joly and Kaufmann, 2008; Rogers-Hayden and Pidgeon, 2007). We found that articles promoting upstream engagement often ascribed this responsibility to policy-makers, rather than scientists.^4^ We do not advocate for a one-size-fits-all approach, and there are certainly circumstances where public involvement at the point of policy-making is sufficient. However, it is worth asking what decisions have been made up to this point determining the research questions posed, methodologies adopted and the technological solutions that have been proposed. What kinds of futures have already been imagined, and what resources are marshalled towards realizing them before publics have been considered?

Stirling’s (2008) conceptualization of knowledge production as a process of ‘opening up’ and ‘closing down’ issues to wider public scrutiny is useful for considering the implications of different imagined publics. Genomics research that opens up deliberations of novel technologies explores alternative paths of action based on a variety of public and scientific values and objectives. Decisions emanating from opened-up processes may be seen as more socially robust, as all options have been on the table rather than foreclosed. At present, decision-making about genomic technologies in forest contexts is narrowly limited to scientific expertise and many views are underrepresented (Studies 30, 34, 35 in Table 1). By constraining the public’s role in the production of knowledge to later stages of knowledge production (e.g. policy development), deficit approaches preclude the possibility of engaging publics that are supposed to benefit from (and may be impacted by) genomics.

Closed-down processes observed in this study are also found to harbour the assumption that adoption is a desirable outcome, prompting some scholars to criticize public engagement being conducted for the sake of selling or promoting the acceptance of science and technology (Corner et al., 2013; Stirling, 2007). We wish to extend this to public perceptions research. Understanding how public view the risks and benefits of scientific endeavours is an important aim. However, public perception studies conducted after a technology becomes a final product are limited in their ability to explore possible alternatives that could increase the relevance and benefits for the people who participate. Moreover, when data about public views is collected with the aim of ensuring buy-in, it raises questions about the role that acceptability studies play in the science-policy interface and whose interests this research serves. For example, we noted that studies that explicitly supported adoption of genomic technologies across both the ‘tools’ and ‘acceptability’ streams tended to make recommendations for how to improve public uptake. Meanwhile, studies employing co-production approaches to public perceptions research expressed more agnostic or critical stances towards genomics. There was a notable lack of co-productive research undertaken by genomicists themselves. If genomic scientists are absent from co-productive research, then research on public perceptions may be co-producing acceptability, rather than supporting more transformative relations between genomic science and society.

Finally, we found that the deficit model is not only something that shapes how scientists understand publics – it also informs how publics are studied. As Stirling (2008) writes, these studies exercise a certain amount of power in ‘. . .choosing focus, partitioning perspectives, engaging stakeholders, recruiting participants, phrasing questions, bounding remits, . . . [and] documenting findings’ (p. 275). Public perceptions research on forest genomics was dominated by quantitative surveys, which often tested public knowledge. Although we agree with Findlater et al. (2020) that large-scale surveys are more generalizable than deliberative methods, such as focus groups and interviews, survey design can embed certain beliefs about publics in ways that ultimately shape study outcomes. For example, narrowly defining studies to simple associations between knowledge and acceptance may artificially elevate the salience of public (lack of) knowledge relative to other empirically supported factors such as trust (Study 11 in Table 1) and culturally held values (Study 9 in Table 1) in shaping public views. These studies may perpetuate the idea that Western science is the sole or most important form of knowledge, marginalizing Indigenous ways of knowing (Latulippe and Klenk, 2020). Moreover, the idea that public opposition presents a risk to be managed further underscores the potential power dynamics at play in who controls the message about technology’s promises.

## 6. Conclusion and future directions

Our objective in this study has been to trace the presence of different ways of imagining publics in peer-reviewed literature. Forest genomics is a field where the rapid emergence of novel technologies is coinciding with a proliferation of research on how public view the acceptability of these interventions. By bringing several commonly cited imagined publics to bear on this literature, we have demonstrated that assumptions about publics shape the stated goals of engaging with publics (e.g., seeking diversity of views and knowledges, or seeking acceptance), what constitutes a ‘problem’ or ‘barrier’ (e.g. public ignorance, conflicting values), and the solutions that are proposed (e.g. one-way education vs. two-way knowledge exchange).

The finding that imagined publics have implications for how public perceptions are studied is a substantial contribution to STS research on expert understandings of the public. In articles that support a deficit model of the public, heavy reliance on surveys may preclude identifying important factors affecting the science-society relationship that have not been previously identified in the literature. It also perpetuates the construction of publics merely as individuals (Welsh and Wynne, 2013) and neglects the relational dynamics that contextualize public views of science. Still, there are opportunities for expanding and deepening knowledge about genomic science and its publics. In the remainder of this article, we propose an agenda for advancing research on public perceptions of science and technology.

### Diversify methodological approaches

Rather than doing away with quantitative surveys, we suggest that the landscape of public perceptions research should be made more methodologically diverse. Qualitative and mixed-methods approaches can complement surveys by identifying new propositions to test, or generate unexpected insights that may challenge taken-for-granted ideas about publics. Recent work in forest genomics has demonstrated the malleability of public preferences, which poses challenges for survey-based research (Studies 9, 10, 11 in Table 1). Thus, the suggestion that scientists can simply convey educational material to publics to improve acceptance, or to assess how publics view this science, is flawed, unless it is accompanied by an attention to social context. Moreover, scientists should be open to bidirectional learning that might challenge their assumptions and results. A dialogue is required that might change public – and scientists’ – perceptions through the process of knowledge exchange.

### Account for relational dynamics

Public perceptions research can be expanded to account for the dynamic interactions between scientists and other public groups. Researchers interested in studying public views of science and technology can adopt a relational lens by reflecting on how they relate to and imagine the public they seek to survey, including assumptions and biases. While co-production may not always be feasible or appropriate, cross-fertilization between different modes of knowledge production can help inform more rigorous scientific research (Klenk et al., 2011). There is also a need for research that studies the relational dynamics that influence public perceptions of science and technology. There has been growing but limited engagement with trust in the literature surveyed here. A vital area for future research is identifying how (dis)trust and relationships between publics, researchers, and technologies change over time, and with what consequences for the social licence of genomics and other fields seeking public acceptance.

There have been recent calls for co-production research to meaningfully engage with Indigenous knowledge systems and recognize the importance of protecting them as separate evolving knowledge frameworks (Chapman and Schott, 2020; Cooke et al., 2021). Latulippe and Klenk (2020) argue that decolonizing co-production requires Western science to ‘make room’ and ‘move over’ for Indigenous knowledge holders and communities to realize the governance value of their knowledge systems. Anderson and Meshake (2019) suggest that reconciliation through research requires that co-researchers prioritize trust-building and kinship. These advances are important for decolonizing genomic science and conducting research in ways that respect Indigenous rights, knowledge systems, and interests in genomic data, and account for histories of transgressions by genomic researchers (Hudson et al., 2020). For Schott et al. (2020), knowledge co-evolution and co-production in genomics work with Inuit partners involved continually revisiting and revising research objectives throughout the research process, including changing the species being studied through genomics in response to community priorities. While co-production work can be time-consuming and may deviate from the goals stated at the outset, this process can improve the benefits of research for communities and contribute to a greater likelihood of understanding of science, mutual benefits and trust.

### (Re)consider the starting point

Research on science and society is not simply about describing public views – it is also about ‘what to do’ about the problem of public engagement with science. Studies of forest genomics have typically centred genomic science and worked outwards to understand how others perceive the risks and benefits of proposed technologies. This default stance has led some to argue that opposition is rooted in failures of public education or political interests. As an alternative, Burns and Medvecky (2018) argue for decentering science as the point of reference, reversing the focus and centreing it on individuals and their lives. A decentered stance begins by asking why science should be relevant for various publics. As a result, the measurement of success in how science is communicated to publics becomes ‘reach’ rather than ‘ratings’. Treating forests – and people’s connections to them – as the starting point and involving publics throughout the process of research and development, would enhance legitimacy and facilitate meaningful public input into the data, methodology, results and implementation of genomic applications.

## Supplemental Material

sj-pdf-1-pus-10.1177_09636625231210453 – Supplemental material for Constructing the public in public perceptions research: A case study of forest genomics Supplemental material, sj-pdf-1-pus-10.1177_09636625231210453 for Constructing the public in public perceptions research: A case study of forest genomics by Valerie Berseth, Jennifer Taylor, Jenna Hutchen, Vivian Nguyen, Stephan Schott and Nicole Klenk in Public Understanding of Science

## Notes

1. While our focus is on experts’ imaginations of publics, we also acknowledge science is similarly being constructed in the process. McNeil (2013) traces the deficit model’s rise and dominance in STS, accompanied by the less frequently recognized ‘diffusion’ model. The diffusion model views science as occupying a privileged position in society, from which ‘pure’ scientific knowledge becomes diluted as it trickles down to society (Cooter and Pumfrey, 1994). The diffusion model is not represented in Figure 1, as our aim is to characterize views of the public and the diffusion model is more centrally focused on the institution of science, but these models can be considered co-constitutive, and mutually reinforcing.

2. Assisted migration uses genomic data to model organic functioning of forests (e.g. growth, development) under future climate scenarios. Trees carrying desirable genetic traits can then be moved to areas where trees are expected to have a positive response to predicted climate patterns.

3. Also referred to as ‘selective breeding’ or ‘genomic selection’, marker-assisted selection involves linking phenotypic (physical) traits of interest to their specific genotypes to accelerate breeding of selected species/individuals (Thavamanikumar et al., 2013).

4. There are other ways of modelling upstream engagement. Wilsdon and Willis (2004), some of the early proponents of upstream engagement, envisioned upstream public involvement as a way to transform the relationship between science and public decision-making.
