# Supplementary material for: Constructing the public in public perceptions research: A case study of forest genomics
Source: Public Underst Sci. 2023 Dec 14;33(4):483–503. doi: 10.1177/09636625231210453 (PMC11056085; doi:10.1177/09636625231210453)
Supplement: sj-pdf-1-pus-10.1177_09636625231210453 – Supplemental material for Constructing the public in public perceptions research: A case study of forest genomics [file sj-pdf-1-pus-10.1177_09636625231210453.pdf]

Constructing the public in public perceptions research: A case study of forest genomics

Valerie Berseth[1,3]*, Jennifer Taylor[2], Jenna Hutchen[3], Vivian Nguyen[3,4], Stephan Schott[5], and Nicole Klenk[2]

[1]Department of Geography and Environmental Studies, Carleton University, Ottawa, Canada; [2]Department of Geography and Planning, University of Toronto, Toronto, Canada; [3]Department of Biology, Carleton University, Ottawa, Canada; [4]Institute of Environmental and Interdisciplinary Science, Carleton University, Ottawa, Canada; [5]School of Public Policy and Administration, Carleton University, Ottawa, Canada; [6]Department of Physical and Environmental Sciences, University of Toronto Scarborough, Toronto, Canada

Supplemental Material

Contents

Table 1. Descriptions and examples of Imagined Publics

Table 2. Summary data for sampled studies.

Table 1. Descriptions and examples of Imagined Publics

Imagined Public	Descriptions	Example Quotes
Uninformed	• Public(s) lack(s) knowledge about or familiarity with science<br>• Public understanding and/or acceptance of science can be improved through education	"The finding that the attitude that it is wrong to "meddle" or "tamper" with nature is so prevalent among UK publics suggests a failure of public education. … This suggests that the development of an effective tree disease policy needs to go hand-in-hand with a broader strategy of public education involving other policy areas dealing with the impacts of accelerated environmental change" (Jepson and Arakelyan, 2017)<br><br>"Our results suggest that greater knowledge of forestry (and indirectly of the increasing impacts of climate change) may translate into greater willingness to act (Bliss et al. 2006)" (Peterson St-Laurent et al., 2018)
Disengaged	• Public(s) lack(s) interest in science and technology in general or in specific areas	"Two conditions could ensure that assisted colonization does not become mired in public contestation: The public could remain unengaged in the controversy, or relevant publics could be enrolled in a discussion of the values underlying conservation such that they feel ownership of whatever decisions are made." (Neff and Larson, 2014)<br><br>"While the survey was not purposefully targeted to avoid non-believers in climate change, the low observed percentage compared to other studies in Canada (Mildenberger et al. 2016) and the USA (Leiserowitz et al. 2016) may be explained by a low response rate to invitations to the survey by non-believers due to lack of interest in the subject (i.e., climate change adaptation)." (Peterson St-Laurent et al., 2018)
Risk	• Concern about controversy and/or the potential for adverse public reaction to science and technology<br>• Public opposition is caused by misinformation, anti-science views, and/or political interests	"However, people tend not to make well-reasoned judgments about emerging technologies, especially in public opinion surveys, which creates substantial challenges for scientists and policy-makers who seek to anticipate and mitigate potential controversies." (Findlater et al., 2020)<br><br>"Although forest products from genetically modified trees might not be in the market soon, marketing claims concerning the non-GM nature of forest products should be administered, as it may upsurge individual's perception of risk." (Maruta et al., 2018)
Co-Producers of Knowledge	• All knowledge production supports particular forms of social order, identities, and relations<br>• Public(s) are valuable actors in the production of knowledge	"Co-production expands discussions about uncertainty beyond awareness of the limitations of knowledge, to encompass recognition of the tacit commitments to particular forms of social order that underpin all knowledge claims. As Brian Wynne (1993, 2005, 2007) has argued in this journal and elsewhere, assessments of uncertainty by scientific experts embed and project assumptions about publics and legitimate areas of public concern, yet often such connections are not made explicit and, hence, are not open to scrutiny and deliberation." (Blue and Davidson, 2020)<br><br>"We join other scholars (e.g. Corner et al., 2012; Stirling, 2007) in inviting researchers and decision-makers to understand the concerns and ethical sensitivities deemed important to different publics, in the hope of incorporating these into policy." (Pelai et al., 2020)

Table 1 continued…

Imagined Public	Descriptions	Example Quotes
Consumers	Public(s) are stakeholders or end-users that would support adoption if genomics provided economic gains or if technologies were cost-effective	"Further, scientifically based information on the expected purchasing behaviour of individuals is an important contribution for use by developers and policy-makers. For the developers, this type of information is imperative, as the anticipation of feasible markets will stimulate investments." (Maruta et al., 2018)<br><br>"It is hypothesized that stakeholders would be supportive of this tool if: it provides significant genetic gains and is thus economically beneficial; it provides seeds that are significantly superior from a risk-reducing perspective (e.g., resistant to pests, diseases, drought); and/or it provides trees with improved wood quality and volume without any genetic modification." (Nilausen et al., 2014)
Owners	• Forest lands and resources are publicly owned<br>• Governments are custodians of forests on behalf of citizens<br>• Specific to Canadian studies	"The importance of public and stakeholder participation is especially salient in jurisdictions where the governments and forest practitioners are the custodians of publicly owned forests." (Peterson St-Laurent et al., 2019)<br><br>"Canada's forests are mostly publically owned and therefore subject to public forest policy with its regulations, legislation and directives." (Porth et al., 2015)

Table 2. Summary data for sampled studies.

	Short Citation	Lead Author Country	Lead Author Affiliation Type[1]	Article Type[2]	Methods	Stream
1	Aubin et al. (2011)	Canada	Government	Research	Literature review	Acceptability
2	Aucott and Parker (2020)	USA	Academic	Essay	None	Tool
3	Bilodeau et al. (2018)	Canada	Government	Review	Literature review	Tool
4	Blue and Davidson (2021)	Canada	Academic	Research	Interviews	Acceptability
5	Ćalić et al. (2016)	Italy, USA	Academic, Government	Review	None	Tool
6	Chaves et al (2021)	Brazil	Academic	Research	None	Tool
7	Crann et al. (2015)	Canada	Academic	Research	Interviews	Acceptability
8	Cullingham et al. (2019)	Canada	Academic	Review	None	Tool
9	Findlater et al. (2022)*	Canada	Academic	Research	Focus group	Acceptability
10	Findlater et al. (2020)	Canada	Academic	Research	Survey	Acceptability
11	Hagerman et al. (2021)	Canada	Academic	Research	Survey	Acceptability
12	Hajjar et al. (2014)	Canada	Academic	Research	Survey	Acceptability
13	Hamelin (2012)	Canada	Academic, Government	Symposium	None	Tool
14	Hamelin and Roe (2020)	Canada	Academic	Review	None	Tool
15	Han et al. (2021)	UK	Academic	Comment	None	Tool
16	Harfouche et al. (2011)	Italy	Academic	Review	None	Tool
17	Hazarika et al. (2021)	Austria	Government	Research	Survey	Acceptability
18	Hewitt et al. (2011)	Canada	Academic	Review	Literature review	Acceptability
19	Homyack et al. (2021)	USA	Industry	Essay	None	Tool
20	Hope et al. (2017)	Canada	Government	Review	None	Tool
21	Huang et al. (2021)	China	Academic	Research	None	Tool
22	Isabel et al. (2019)	Canada	Academic, Government	Perspective	None	Tool
23	Jepson and Arakelyon (2017)	UK	Academic	Research	Survey, Focus Group	Acceptability
24	Kerio et al. (2020)	USA	Academic	Review	None	Tool
25	Lavrik (2021)	China	Academic	Review	None	Acceptability
26	Maruta et al. (2018)	Canada	Academic	Research	Survey	Acceptability
27	Moshofsky et al. (2019)	Canada	Academic	Research	Focus group	Acceptability
28	Neale (2007)	USA	Academic, Government	Review	None	Tool
29	Neale and Kremer (2011)	USA, France	Academic	Review	None	Tool
30	Neff and Larson (2014)	Canada	Academic	Research	Survey (Q-method)	Acceptability
31	Nilausen et al. (2016)	Canada	Academic	Proposed Research	Interviews, survey	Acceptability
32	Nilausen et al. (2014)	Canada	Academic	Research	Interviews, survey	Acceptability
33	Pelai et al. (2021)	Canada	Academic	Research	Interviews	Acceptability
34	Pelai et al. (2021)	Canada	Academic	Research	Interviews	Acceptability
35	Pelai et al. (2020)	Canada	Academic	Review	Literature review	Acceptability
36	Peterson St-Laurent et al. (2021a)	Canada	Academic	Review	Literature review	Acceptability
37	Peterson St-Laurent et al. (2021b)	Canada, Switzerland	Academic, Intergov. Organization	Research	Survey	Acceptability
38	Peterson St-Laurent et al. (2019)	Canada	Academic	Research	Survey	Acceptability
39	Peterson St-Laurent et al. (2018)	Canada	Academic	Research	Survey	Acceptability

Table 2 continued…

	Short Citation	Lead Author Country	Lead Author Affiliation Type[1]	Article Type[2]	Methods	Stream
40	Plomion et al. (2016)	France	Academic, Research Institute	Review	None	Tool
41	Porth et al. (2015)	Canada	Academic	Review	None	Tool
42	Sork et al. (2013)	USA	Academic	Opinion	None	Tool
43	Thiffault et al. (2021)	Canada	Academic, Government	Review	Survey	Acceptability
44	Touchette et al. (2021)	Canada	Academic, Government	Review	None	Acceptability

[1] Article contributions by country and the organization affiliation of the lead author. “Academia” refers to any post-secondary institution. “Government” refers to any agency, ministry, or department associated with a provincial, state, territorial, or federal government. “Industry” refers to a for-profit organization.

[2] Articles included in this review organized by contribution type. “Research” refers to primary peer-reviewed literature. “Review” is inclusive of all secondary literature review types (e.g. narrative reviews, meta-analysis, systematic reviews).

* Paper was published OnlineFirst in 2021 at the time of data collection. The publication date has since been revised by the journal to 2022. We cite the revised date of publication in this table and the reference list, but the paper has been included in Figure 3 (main body) according to the original date of publication to avoid confusion, as our search was limited to publications prior to 2022.
